# Supplementary material for: Drosophila SPG12 ortholog, reticulon-like 1, governs presynaptic ER organization and Ca2+ dynamics
Source: J Cell Biol. 2023 Mar 23;222(6):e202112101. doi: 10.1083/jcb.202112101 (PMC10072275; doi:10.1083/jcb.202112101)
Supplement: Table S2 — shows crosses used in this work. [file JCB_202112101_TableS2.docx]

**Supplementary Table 2. Crosses used in this work.** The table shows simplified genotypes of the F0 parental stocks (from **Supp. Table 1**) used to generate the F1 larvae shown or analyzed in figures, as well as the F1 summary genotypes used. Floating balancers are shown in brackets. Where possible we selected non-balancer homozygous parents, otherwise we counterselected against the dominant balancer markers in F1 larvae: Tubby (on *CyO, Tb* or on *TM6B*) or the widely expressed GFP of *CyO::GFP*. Normally two alternative crosses were performed, one with both parents *WT (+)*, the other with both parents mutant for *Rtnl1*; the alternative alleles used are in brackets in F0 parental genotypes. In F1 larvae, the designation *Rtnl1^x^* represents any of the *Rtnl1* genotypes possible in the crosses shown.

| **Fig** | **F0 parent stock 1** | **F0 parent stock 2** | **F1 Genotype** |
| --- | --- | --- | --- |
| 2A, S3 | *( + OR Rtnl1^18^); Ib-GAL4, UAS-CD4::tdGFP / (TM6B)* | *(+ OR Rtnl1^18^) / (CyO::GFP); UAS-tdTom::Sec61β / (TM6B)* | *Rtnl1^x^; Ib-GAL4, UAS-CD4::tdGFP / UAS-tdTom::Sec61β* |
| 2B | *( + OR Rtnl1^18^) ; Is-GAL4, UAS-CD4::tdGFP / (TM6B)* | *( + OR Rtnl1^18^)/(CyO::GFP); UAS-tdTom::Sec61β/(TM6B)* | *Rtnl1^x^; Is-GAL4, UAS-CD4::tdGFP / UAS-tdTom::Sec61β* |
| 2C | One of:  *- ( + OR Rtnl1^18^); + / (TM6B)*  *- ( + OR Rtnl1^18^); UAS-Rtnl1::HA / (TM6B)* | *( + OR Rtnl1^18^) / (CyO::GFP); D42-GAL4, UAS-tdTom::Sec61β / (TM6B)* | *Rtnl1^x^; D42-GAL4, UAS-tdTom::Sec61β / (+ OR UAS-Rtnl1::HA)* |
| 2D-E, S3 | *( + OR Rtnl1^18^)* | *( + OR Rtnl1^18^) / (CyO, Tb); Ib-GAL4, UAS-Sturkopf::GFP / (TM6B)* | *Rtnl1^x^; Ib-GAL4, UAS-Sturkopf::GFP / +* |
| 3B-E, S5C | *( + OR Rtnl1^18^);*  *Ib-GAL4, UAS-CD4::tdTom / (TM6B)* | *( + OR Rtnl1^18^) / (CyO::GFP);*  *UAS-GFP::HDEL / (TM6B)* | *Rtnl1^x^;*  *Ib-GAL4,*  *UAS-CD4::tdTom / UAS-GFP::HDEL* |
| 4 | *( + OR Rtnl1^18^) / (CyO, Tb);*  *Ib-GAL4, UAS-Sturkopf::GFP / (TM6B)* | *( + OR Rtnl1^18^) / (CyO, Tb);*  *Ib-GAL4,*  *UAS-STIM::mCherry / (TM6B)* | *Rtnl1^x^;*  *Ib-GAL4, UAS-Sturkopf::GFP / UAS-STIM::mCherry* |
| S4A | *( + OR Rtnl1^18^)* | *( + OR Rtnl1^18^) / (CyO, Tb); D42-GAL4, UAS-tdTom::Sec61β, UAS-CD4::tdGFP / (TM6B)* | *Rtnl1^x^; D42-GAL4, UAS-tdTom::Sec61β, UAS-CD4::tdGFP / +* |
| S4B | *( + OR Rtnl1^18^)* | *(Rtnl1^+^ OR Rtnl1^1^) / (CyO, Tb); D42-GAL4, UAS-tdTom::Sec61β / (TM6B)* | *Rtnl1^x^ ; D42-GAL4, UAS-tdTom::Sec61β / +* |
|  | *(Rtnl1^+^ OR Rtnl1^1^) / (CyO, Tb); D42-GAL4, UAS-Rtnl1::GFP / (TM6B)* | *(Rtnl1^+^ OR Rtnl1^18^) / (CyO::GFP); UAS-tdTom::Sec61β / (TM6B)* | *Rtnl1^x^; D42-GAL4, UAS-Rtnl1::GFP / UAS-tdTom::Sec61β* |
| S5A | *Rtnl1^+^ / (CyO::GFP); UAS-GFP::HDEL / (TM6B)* | *UAS-tdTom::Sec61β / (CyO, Tb); Ib-GAL4 / (TM6B)* | *Rtnl1^+^ / UAS-tdTom::Sec61β; UAS-GFP::HDEL / Ib-GAL4* |
| S5B | *(Rtnl1^+^) / (CyO::GFP) OR Rtnl1^1^ /*  *(CyO, Tb); UAS-GFP::HDEL / (TM6B)* | *(Rtnl1^+^ OR Rtnl1^18^) / (CyO, Tb); Ib-GAL4, UAS-CD4::tdGFP / (TM6B)* | *Rtnl1^x^; UAS-GFP::HDEL / Ib-GAL4, UAS-CD4::tdGFP* |
| S6 | *(Rtnl1^+^ OR Rtnl1^18^);*  *Ib-GAL4, UAS-CD4::tdTom / (TM6B)* | *(Rtnl1^+^ OR Rtnl1^18^);*  *UAS-PLC𝛿::PH::GFP / (TM6B)* | *Rtnl1^x^;*  *Ib-GAL4, UAS-CD4::tdTom / UAS-PLC𝛿::PH::GFP* |
| S7, S8 | *(Rtnl1^+^ OR Rtnl1^18^);*  *Is-GAL4, Mhc-SynapGCaMP6f / (TM6B)* | *(Rtnl1^+^ OR Rtnl1^18^) / (CyO::GFP); UAS-tdTom::Sec61β / (TM6B)* | *Rtnl1^x^; Is-GAL4,*  *Mhc-SynapGCaMP6f / UAS-tdTom::Sec61β* |
| 6, S11 | *(Rtnl1^+^ OR Rtnl1^18^) / (CyO::GFP); Is-GAL4, UAS-ER-GCaMP6-210 / (TM6B)* | *(Rtnl1^+^ OR Rtnl1^18^) / (CyO::GFP); UAS-tdTom::Sec61β / (TM6B)* | *Rtnl1^x^; Is-GAL4,*  *UAS-ER-GCaMP6-210 / UAS-tdTom::Sec61β* |
| 7, S11 | *(Rtnl1^+^ OR Rtnl1^18^) / (CyO::GFP); Ib-GAL4, UAS-ER-GCaMP6-210 / (TM6B)* | *(Rtnl1^+^ OR Rtnl1^18^) / (CyO::GFP); UAS-tdTom::Sec61β / (TM6B)* | *Rtnl1^x^; Ib-GAL4,*  *UAS-ER-GCaMP6-210 / UAS-tdTom::Sec61β* |
| 5 | *(Rtnl1^+^ OR Rtnl1^18^) / (CyO,Tb); / UAS-tdTom-p2a-GCaMP56 / (TM6B)* | *(Rtnl1^+^ OR Rtnl1^18^)/(CyO,Tb); / Ib-Gal4 / (TM6B)* | *Rtnl1^x^; UAS-tdTom-p2a-GCaMP56/ Ib-GAL4* |
| 5, S10 | *(Rtnl1^+^ OR Rtnl1^18^) ; Ib-GAL4,*  *UAS-myr::GCaMP6s / (TM6B)* | *(Rtnl1^+^ OR Rtnl1^18^) / (CyO::GFP); UAS-tdTom::Sec61β / (TM6B)* | *Rtnl1^x^; UAS-tdTom::Sec61β / Ib-GAL4,*  *UAS-myr::GCaMP6s* |
| 5, S9 | *(Rtnl1^+^ OR Rtnl1^18^) / (CyO,Tb); Is-GAL4, UAS-myr::GCaMP6s / (TM6B)* | *(Rtnl1^+^ OR Rtnl1^18^) / (CyO::GFP); UAS-tdTom::Sec61β/(TM6B)* | *Rtnl1^x^; UAS-tdTom::Sec61β / Is-GAL4,*  *UAS-myr::GCaMP6s* |
| 5E | *(Rtnl1^+^ OR Rtnl1^18^) ; Ib-GAL4,*  *UAS-myr::GCaMP6s / (TM6B)* | *(Rtnl1^+^ OR Rtnl1^18^) / (CyO,Tb); + / (TM6B)* | *Rtnl1^x^; Ib-GAL4,*  *UAS-myr::GCaMP6s / +* |
| 5E | *(Rtnl1^+^ OR Rtnl1^18^) ; Ib-GAL4,*  *UAS-myr::GCaMP6s / (TM6B)* | *(Rtnl1^+^ OR Rtnl1^18^) / (CyO,Tb); UAS-Rtnl1::HA / (TM6B)* | *Rtnl1^x^; Ib-GAL4,*  *UAS-myr::GCaMP6s / UAS-Rtnl1::HA* |
| S9 | *(Rtnl1^+^ OR Rtnl1^18^) / (CyO,Tb); Is-GAL4, UAS-myr::GCaMP6s / (TM6B)* | *(Rtnl1^+^ OR Rtnl1^18^) / (CyO::GFP); UAS-tdTom::Sec61β / (TM6B)* | *Rtnl1^+^ / Rtnl1^18^; UAS-tdTom::Sec61β /*  *Is-GAL4, UAS-myr::GCaMP6s* |
| S9 | *Rtnl1^18^ / (CyO,Tb); Is-GAL4,*  *UAS-myr::GCaMP6s / (TM6B)* | *Rtnl1^1^ / (CyO::GFP); UAS-tdTom::Sec61β / (TM6B)* | *Rtnl1^1^ / Rtnl1^18^; UAS-tdTom::Sec61β / Is-GAL4, UAS-myr::GCaMP6s* |
| S13 | *(Rtnl1^+^ OR Rtnl1^18^) ; Ib-GAL4,*  *UAS-CEPIA3mt::myc / (TM6B)* | *(Rtnl1^+^ OR Rtnl1^18^) ; Ib-GAL4, UAS-CEPIA3mt::myc / (TM6B)* | *Rtnl1^x^; Ib-GAL4, UAS-CEPIA3mt::myc* |
| 8, S13 | *(Rtnl1^+^ OR Rtnl1^18^) ; Ib-GAL4,*  *UASTattB-CEPIA3mt / (TM6B)* | *(Rtnl1^+^ OR Rtnl1^18^) / (CyO::GFP); UAS-tdTom::Sec61β / (TM6B)* | *Rtnl1^x^; UAS-tdTom::Sec61β / Ib-GAL4,*  *UAS-CEPIA3mt* |
| 8, S12 | *(Rtnl1^+^ OR Rtnl1^18^) / (CyO,Tb); Is-GAL4, UAS-CEPIA3mt/ (TM6B)* | *(Rtnl1^+^ OR Rtnl1^18^) / (CyO::GFP); UAS-tdTom::Sec61β/ (TM6B)* | *Rtnl1^x^; UAS-tdTom::Sec61β / Is-GAL4,*  *UAS-CEPIA3mt* |
| S12 | *(Rtnl1^+^ OR Rtnl1^18^) / (CyO,Tb); Is-GAL4, UAS-CEPIA3mt / (TM6B)* | *(Rtnl1^+^ OR Rtnl1^18^ ) / (CyO::GFP); UAS-tdTom::Sec61β/(TM6B)* | *Rtnl1^+^/Rtnl1^18^; UAS-tdTom::Sec61β / Is-GAL4, UAS-CEPIA3mt* |
| S12 | *Rtnl1^18^ / (CyO,Tb);*  *Is-GAL4, UAS-CEPIA3mt / (TM6B)* | *Rtnl1^1^/(CyO::GFP); UAS-tdTom::Sec61β / (TM6B)* | *Rtnl1^1^ / Rtnl1^18^; UAS-tdTom::Sec61β / Is-GAL4, UAS-CEPIA3mt* |
| 8E | *(Rtnl1^+^ OR Rtnl1^18^) ;*  *Ib-GAL4, UAS-CEPIA3mt / (TM6B)* | *(Rtnl1^+^ OR Rtnl1^18^) /(CyO,Tb);*  *+/(TM6B)* | *Rtnl1^x^; Ib-GAL4, UAS-CEPIA3mt::myc / +* |
| 8E | *(Rtnl1^+^ OR Rtnl1^18^) ;*  *Ib-GAL4, UAS-CEPIA3mt / (TM6B)* | *(Rtnl1^+^ OR Rtnl1^18^) / (CyO,Tb); UAS-Rtnl1::HA / (TM6B)* | *Rtnl1^x^; Ib-GAL4, UAS-CEPIA3mt::myc / UAS-Rtnl1::HA* |
