# Supplementary figures and images for: Drosophila SPG12 ortholog, reticulon-like 1, governs presynaptic ER organization and Ca2+ dynamics
Source: J Cell Biol. 2023 Mar 23;222(6):e202112101. doi: 10.1083/jcb.202112101 (PMC10072275; doi:10.1083/jcb.202112101)

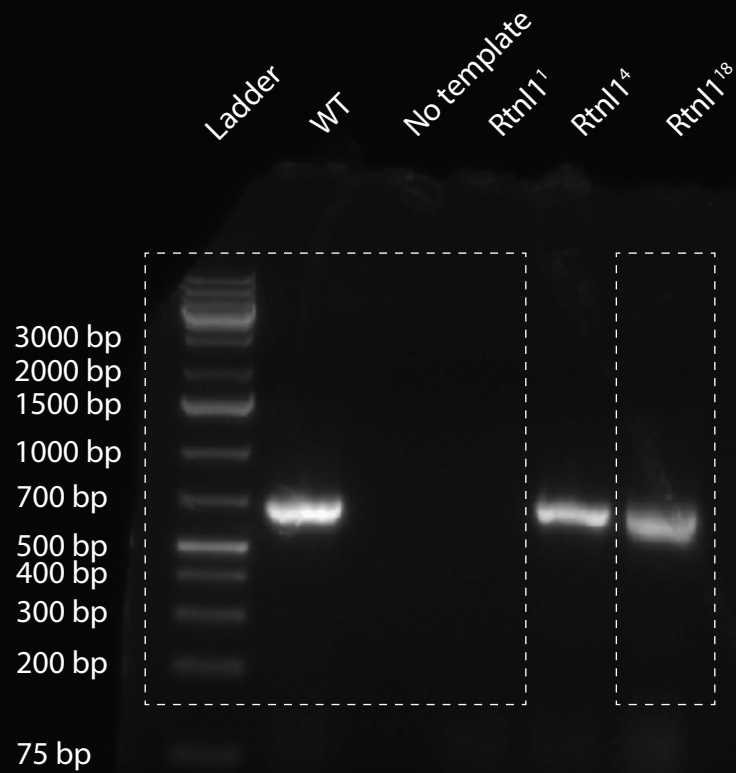

Supplement: SourceData FS2 — is the source file for Fig. S2. [file JCB_202112101_SourceDataFS2.pdf]
